# Pure Motor Stroke Secondary to Cerebral Infarction of Recurrent Artery of Heubner after Mild Head Trauma: A Case Report

**DOI:** 10.3889/oamjms.2016.013

**Published:** 2016-01-08

**Authors:** Ali Yilmaz, Zahir Kizilay, Ayca Ozkul, Bayram Çirak

**Affiliations:** 1 *Adnan Menderes University, Faculty of Medicine, Neurosurgery Department, Aydin, Turkey*; 2 *Adnan Menders University, Faculty of Medicine, Department of Neurology, Aydin, Turkey*; 3 *Pamukkale University, Faculty of Medicine, Department of Neurosurgery, Denizli, Turkey*

**Keywords:** childhood stroke, Heubner artery, post-traumatic stroke, head trauma

## Abstract

**BACKGROUND:**

The recurrent Heubner’s artery is the distal part of the medial striate artery. Occlusion of the recurrent artery of Heubner, classically contralateral hemiparesis with fasciobrachiocrural predominance, is attributed to the occlusion of the recurrent artery of Heubner and is widely known as a stroke syndrome in adults. However, isolated occlusion of the deep perforating arteries following mild head trauma also occurs extremely rarely in childhood.

**CASE REPORT:**

Here we report the case of an 11-year-old boy with pure motor stroke. The brain MRI showed an acute ischemia in the recurrent artery of Heubner supply area following mild head trauma. His fasciobrachial hemiparesis and dysarthria were thought to be secondary to the stretching of deep perforating arteries leading to occlusion of the recurrent artery of Heubner.

**CONCLUSION:**

Post-traumatic pure motor ischemic stroke can be secondary to stretching of the deep perforating arteries especially in childhood.

## Introduction

The recurrent artery of Heubner (RAH), the junction of the anterior cerebral artery and ACoA or distal A1 segment coursing backward along the A1 segment, arises as a single vessel from the portion near to the anterior communicating artery (ACoA) [1]. It supplies the head of the caudate nucleus, the anterior inferior part of the internal capsule’s anterior limb, the anterior globus pallidus and putamen and the anterior thalamus, and some parts of the uninate fasciculus, olfactory region and the hypothalamus [2]. Classically, contralateral hemiparesis with fasciobrachiocrural predominance is attributed to occlusion of the RAH and is widely known as stroke syndrome in adults [3, 4]. However, isolated occlusion of the deep perforating arteries following mild head trauma occurs extremely rarely in childhood.

Here we report the case of an 11-year-old boy with a pure motor stroke secondary to an acute ischemic infarction at the RAH supply area immediately following mild head trauma.

## Case presentation

**Figure 1:**
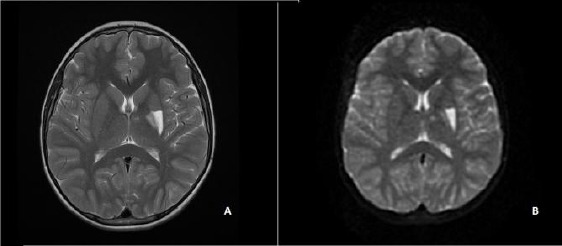
*The cerebral MRI showing a hyperintense lesion at the left putamen and globus pallidus interna and externa on T2 (A) and diffusion weighted images (B)*.

An 11-year-old boy was brought to the emergency room (ER) because of difficulty in speech and weakness on his right arm, which appeared soon after he hit his head upon falling from his bicycle. He was not unconscious but 20 minutes after falling difficulty in speech and right arm paresis was seen. In his initial neurological examination GCS was 14 and his pupils were reactive to light and isochoric. He had dysarthria and right fasciobrachial hemiparesis with muscle strength of 4/5. The brain CT revealed neither skull fracture nor hemorrhagia of the intra-/extra-axial structures. A brain MRI after 24 hours showed a hyperintense lesion at the left putamen and globus pallidus interna and externa on T2 weighted images (Figure 1A), and diffusion restriction was also seen at the aforementioned structures consistent with the RAH supply area (Figure-1B). His cerebral and carotid MR angiography revealed no pathology and his parents did not give permission for cerebral angiography, and so this could not be performed. Isolated post-traumatic occlusion of the perforating arteries is extremely rare in children. Therefore, a large scale laboratory assessment was performed to exclude coexisting systemic vasculitic diseases, emboligenic heart diseases and haematological disorders. Erythrocyte sedimentation rate, CRP, platelet count, PT, PTT, antithrombin III, homocycteine, von Willebrand factor, activated protein C resistance, and protein C and S assays were all normal. Lupus anticoagulants, anticardiolipin and antiphospholipid antibodies were negative as were antinuclear and anti-ds DNA antibody titers. He was also assessed for ophthalmological and dermatological manifestations of the vasculitic diseases, but these revealed negative results.

During the hospitalization period he did not develop any further neurological deficits or convulsive episodes. A physical rehabilitation program was applied and he was discharged ten days later. The latest neurological examination after discharge was performed six months later and he had full muscle strength on the right side. Apart from the possibility of a mild cognitive dysfunction, his school performance was acceptable.

## Discussion

Moderate to severe head injuries in children can lead to skull fractures, shearing injury or infarct/oedema [5, 6]. The majority of cerebral artery occlusions occur in the anterior and middle cerebral arteries due to vasospasm, embolus, thrombus or dissecting aneurisms. There also may be traumatic dissection of the carotid arteries or vessels of the circle of Willis as well as congenital predisposition to rupture of intracranial arteries. However, cerebral ischemic lesions are extremely rare in childhood with an incidence of 0.2-0.63/100 000 per year [3, 7]. Less than 2% of all ischemic strokes in childhood occur in the basal ganglia after mild head trauma, which is attributed to the vasospasm of the deep perforating arteries. Most pure motor ischaemic strokes are due to small arterial vessel disease in old patients with a mean (SD) age of 72.2 years [8, 9]. In 15%, they may be associated with other stroke subtypes [10].

The deep perforators of the carotid system refers to the anterior choroidal artery and posterior communicating artery, the lateral lenticulostriate arteries that originate from the middle cerebral artery and the medial lenticulostriate arteries and the RAH that originates from the anterior cerebral artery [1, 2, 7, 9, 11, 12]. Unlike the small penetrating arteries, selective occlusion of the main trunk of either the RAH or lenticulostriate arteries may result from vasospasm or thromboembolism following head injury, probably because of the anatomical characteristics of these arteries in children. The occlusion of the lenticulostriate, thalamoperforating, or choroideal arteries may result in basal ganglionic infarction after head injuries [13].

According to a recent report history, mild head trauma is one of the most frequent risk factors for ischemic stroke of basal ganglia in children [8]. Several mechanisms of arterial stroke have been proposed. Mechanical disruption of the flow in the perforating branches or intimal trauma and subsequent thrombosis can lead to occlusion as well as transient arterial spasm induced by trauma [8].

Although the affected area is relatively small, this can cause contralateral hemiparesis with fasciobrachiocrural predominance. Together with this, dysarthria, epilepsy, athetosis, and cognitive and behavioural disturbances are reported [11]. Sometimes it may be hard to attribute these clinical findings to minor head trauma without loss of consciousness. Therefore, differential diagnostic assessment is important. Assessment of common risk factors of cerebral infarctions of adults, imaging and laboratory work should be directed to determine secondary causes such as traumatic dissection of the common carotid and internal carotid arteries or vessels of the circle of Willis, congenital predisposition to rupture of the intracranial arteries, heart disease leading to embolus formation and congenital thrombophilia. Although cranial MRI showed the infarction of the RAH supply area in our patient, a cerebral angiography or CT angiography would have been informative and excluded arterial dissection if it had been performed. Moreover, laboratory assessment to differentiate systemic diseases leading to vasculitis or prothrombotic states revealed normal test results.

We concluded that post-traumatic pure motor ischemic stroke can be secondary to stretching of the deep perforating arteries during hemispheric movement at a perpendicular angle, leading to occlusion of RAH.

## References

[1] Perlmutter D, Rhoton AL (1978). Microsurgical anatomy of the distal anterior cerebral artery. Journal of Neurosurgery.

[2] Ghika JA, Bogousslavsky J, Regali F (1990). Deep perforators from the carotid system:template of the vascular territories. Arch Neurol.

[3] Critchley M (1930). The anterior cerebral artery and its syndromes. Brain.

[4] Dunker PO, Harris AB (1976). Surgical anatomy of the proximal anterior cerebral artery. Journal of Neurosurgery.

[5] Bagley LJ (1999). Imaging of neurological emergencies:trauma, hemorrhage, and infarction. Semin Roentgenol.

[6] Baumgartner J, Fletcher JM, Alpert B (2000). Acute neuroradiologic findings in young children with inflicted or noninflicted traumatic brain injury. Childs Nerv Syst.

[7] Weiller C, Ringelstein EB, Reiche W, Thron A, Buell U (1990). The large striatocapsular infarct. A clinical and pathophysiological entity. Arch Neurol.

[8] Buompadre MC, Arroyo HA (2009). Stroke Group. Basal ganglia and internal capsule stroke in childhood- risk factors, neuroimaging and outcome in a series of 28 patients:A tertiary hospital experience. J Child Neurol.

[9] Toole JF, Burrow DD, Youmans JR (1990). Pathophysiology and clinical evaluation of ischemic vascular disease. Neurological surgery.

[10] Arboix A, Padilla I, Massons J, Garcia-Eroles L, Comes E, Targa C (2001). Clinic study of 222 patients with pure motor stroke. J Neurol Neurosurg Psychiatry.

[11] Gomes E, Dujovny M, Umansky F, Ausman JI, Diaz FG, Ray WJ (1984). Microsurgical anatomy of the recurrent artery of Heubner. Journal of Neurosurgery.

[12] Umansky F, Gomez FB, Dujovny M, Diaz FG, Ausman JI, Mirchandani HG, Berman SK (1984). The perforating branches of middle cerebral artery. A microsurgical study. Neurosurg.

[13] Osborn AG, Osborn AG (1994). Craniocerebral trauma. Diagnostic euroradiology.

